# Efficacy and safety of anisodine hydrobromide injection for acute ischemic stroke: a systematic review and meta-analysis

**DOI:** 10.3389/fphar.2023.1290755

**Published:** 2023-11-15

**Authors:** Yang Wang, Feng Wan, Peiqun Hu, Benxiang He, Yushi Hu, Yunlu Liu

**Affiliations:** ^1^ School of Sports Medicine and Health, Chengdu Sport University, Chengdu, Sichuan, China; ^2^ Postdoctoral Workstation, Affiliated Sport Hospital of Chengdu Sport University, Chengdu, Sichuan, China; ^3^ State Key Laboratory of Southwestern Chinese Medicine Resources, Chengdu University of Traditional Chinese Medicine, Chengdu, Sichuan, China; ^4^ Sichuan Academy of Chinese Medicine Science, Chengdu, Sichuan, China; ^5^ Institute of Laboratory Animal Sciences, Sichuan Provincial People’s Hospital, University of Electronic Science and Technology of China, Chengdu, Sichuan, China

**Keywords:** acute ischemic stroke, anisodine hydrobromide injection, systematic review, meta-analysis, efficacy

## Abstract

**Background:** Acute ischemic stroke (AIS) is a leading cause of death and disability worldwide. This study aimed to evaluate the efficacy and safety of anisodine hydrobromide (Ani) injection in the treatment of AIS.

**Methods:** Randomized controlled trials (RCTs) based on Ani injection for the treatment of AIS were retrieved from both Chinese and English databases. The retrieval period was from the databases’ inception to May 2023. The Cochrane Collaboration Risk of Bias Tool was used to assess the methodological quality. The outcome indicators were analyzed using RevMan 5.3 software.

**Results:** We included the findings of 11 RCTs encompassing 1,337 patients with AIS. Our meta-analysis revealed that Ani injection supplementation significantly reduced the National Institutes of Health Stroke Scale [MD = −1.53, 95%CI = (−1.94, −1.12), *p* < 0.00001], modified Rankin Scale [MD = −0.89, 95%CI = (−0.97, −0.81), *p* < 0.00001], and the relative time to peak [SMD = −0.81, 95%CI = (−1.08, −0.55), *p* < 0.00001] significantly. Additionally, Ani injection significantly increased the Barthel Index [MD = 10.65, 95%CI = (4.30, 17.00), *p* = 0.001], relative cerebral blood volume [SMD = 0.28, 95%CI = (0.02, 0.53), *p* = 0.03], and clinical efficacy [RR = 1.2, 95%CI = (1.08, 1.34), *p* = 0.001]. No statistically significant difference in the rate of adverse events was observed between the Ani injection supplemental group and the control group.

**Conclusion:** Based on currently published evidence, Ani injection was found to be effective and safe in improving AIS outcome. Nevertheless, limitations of the included RCTs still exist, and thus, more multi-center, large-sample, high-quality RCTs are required to further verify the efficacy and safety of Ani injection in patients with AIS.

**Systematic Review Registration:** [https://www.crd.york.ac.uk/prospero/display_record.php?ID=CRD42023427591], identifier [PROSPERO 2023 CRD42023427591].

## 1 Introduction

Acute ischemic stroke (AIS) is characterized by ischemia, hypoxic necrosis, and softening of the brain tissue due to a sudden interruption of the cerebral blood supply with inadequate collateral circulation, resulting in a series of symptoms of neurological dysfunction (Zhang et al., 2020). AIS is the most common type of cerebral stroke, accounting for approximately 70% of all strokes. Worldwide, AIS is a leading cause of death and disability (Wang et al., 2019). In China, the mortality rate of hospitalized AIS patients within 1 month of onset is approximately 2.3%–3.2%, the 1-year mortality rate after onset is 14.4%–15.4%, and the disability rate is 33.4%–33.8% (Wang et al., 2013; Wang et al., 2017). The associated socioeconomic burden of AIS is huge; for example, the annual expenditure related to AIS, including long-term rehabilitation and unemployment, is estimated to be £ 25.6 billion in the United Kingdom (Robert et al., 2020). Therefore, AIS has become a major global health concern.

At present, regular treatment of AIS consists of a multidisciplinary approach. Treatment management for AIS includes drug therapy, limb rehabilitation, language training, psychological rehabilitation, and health education (Trialists’Collaboration, 2013). Intravenous thrombolysis with recombinant tissue-type plasminogen activator (rtPA) and endovascular therapy have been the mainstay treatments for AIS in recent years (Powers et al., 2018). Both therapeutic strategies aim to rescue ischemic brain tissue with viable potential by recanalization of occluded cerebral arteries and reperfusion of the ischemic penumbra (Robert et al., 2020). Nevertheless, the number of patients with AIS who are eligible for such reperfusion strategies remains low due to the narrow time window of reperfusion therapy (Rodrigues et al., 2016; Bhaskar et al., 2018). More specifically, the therapeutic effect is heavily time dependent; therefore, the stroke symptom onset should be recorded accurately as a clock time to avoid treatment failure. Intravenous thrombolysis and endovascular thrombectomy for AIS patients with an unclear onset time require further exploration (Qiang et al., 2017). Furthermore, clinical evidence has shown that only patients with large vessel occlusive-type AIS are candidates for endovascular therapy, which accounts for less than 20% of AIS cases (Yasha et al., 2019). Symptomatic intracranial hemorrhage after thrombolysis and endovascular treatment in patients with AIS is a major complication that is associated with a devastating clinical outcome. The high frequency of intracranial hemorrhage poses a huge challenge to the clinical management of AIS (Seet and Rabinstein, 2012; Hao et al., 2017). In addition, as a serious complication of vascular recanalization, ischemia–reperfusion injury in the setting of cerebral ischemia following vascular restoration occurs because of a complex series of events, which can evoke parenchymal brain damage (Nour et al., 2013). Therefore, novel therapeutic strategies are urgently required to improve the efficacy and safety of AIS treatment.

A tenet of traditional folk medicine in China is that herbs possess the ability to treat various diseases. Modern researchers have demonstrated that compounds in these medicinal herbs, which consist of multiple ingredients, have multiple pharmacological actions, which are compatible with the complex pathogenesis of diverse human diseases (Chen et al., 2022). For many years in China, various traditional folk herbs have been applied in the treatment of AIS based on the theory of promoting blood circulation and removing blood stasis (Gong and Sucher, 2002). In-depth studies have elucidated the underlying mechanisms of the therapeutic effect of traditional medicinal herbs, which involve the inhibition of excitotoxicity, inflammation, oxidative damage, ionic imbalances, apoptosis, and so on, in the pathophysiological process of AIS (Sucher, 2006). A meta-analysis including 191 clinical trials involving 22 types of traditional Chinese medicine has demonstrated the improvement of neurological deficits after administration (Wu et al., 2007).

*Anisodus tanguticus* (Maxim.) Pascher, also named “Tang Chuan Na Bao” in Ethnologue, one of the indigenous Chinese ethnological plants of the Solanaceae, is mainly grown in the Qinghai–Tibet Plateau (Liu et al., 2005). In traditional Chinese medical theory, *A. tanguticus* possesses the traditional characteristics of nature of a warm, bitter flavor and functions to activate the blood to remove stasis (Chen et al., 2022). Anisodine, a tropane alkaloid extracted from the root of *A. tanguticus*, has been used as an ingredient in the compound preparation for treating ischemic stroke in China for more than a decade due to its significant properties of vasoactivity and improvements in microcirculation. To improve the chemical instability, researchers have developed a hydrobromide form of anisodine (Liu et al., 2020). Recently, anisodine hydrobromide (Ani) injection has been used in the clinical setting for the treatment of AIS in China. Multiple clinical studies have demonstrated the neuroprotective effect of Ani in AIS, which can not only alleviate neurological impairment and reduce dependency in activities of daily living but also improve the cerebral collateral circulation and increase cerebral tissue blood flow perfusion in ischemic areas (Zou et al., 2018; Zhang, 2022). Basic research has revealed that, as a central muscarinic cholinergic receptor blocker, the neuroprotective and cerebral circulation-promoting effect of Ani injection in the treatment of AIS can be correlated to the pharmacological actions of anti-oxidative damage, anti-inflammation, inhibition of neuronal apoptosis, and amelioration of hemorheological changes through regulation of the nitric oxide synthase system, preventing Ca^2+^ influx, decreasing IL-6 serum levels, and modulating angiogenic factors. Furthermore, the ability of Ani to activate the ERK1/2 signaling pathway and regulate ATPase activity is also a key underlying mechanism of action (Chen et al., 2017; Wang et al., 2017; Chen et al., 2017d; Xu et al., 2020; Zeng et al., 2021).

The impact of Ani injection on patients with AIS has been investigated in many clinical trials. In 2021, the earliest meta-analysis conducted by Wang et al. (2021) reported that Ani may have a positive effect in the treatment of ischemic stroke. However, the subjects included in Wang’s study were patients with ischemic stroke at various stages, including both the acute stage and the convalescent stage. In addition, the study objective of several included randomized controlled trials (RCTs) focused on the synergistic effect of Ani combined with acupuncture or butylphthalide. There were certain limitations without further assessment targeting each specific clinical stage (including the acute stage of ischemic stroke) and the pure effect of Ani injection. Therefore, the present study aimed to systematically collect the current clinical evidence regarding Ani injection in the treatment of the acute stage of ischemic stroke and, more specifically, evaluate its efficacy and safety. We hope this meta-analysis and systematic review will provide an accurate and reliable evidence-based reference for its rational use in the clinic.

## 2 Methods

### 2.1 Study registration

This meta-analysis was performed in strict accordance with the Preferred Reporting Items for Systematic Reviews and Meta-Analyses (PRISMA) and was registered in PROSPERO (CRD42023427591).

### 2.2 Search strategy

Both English databases (including EMBASE, PubMed, Cochrane library, and Web of Science) and Chinese databases (including CNKI, VIP, Wanfang, and Chinese Biomedical Literature Database) were searched comprehensively from the date of their respective inception to May 2023 for the identification of eligible data. The following terms used in the search are a combination of MESH terms and free-text words: (“Anisodine hydrobromide injection” (Text word) OR “Anisodine hydrobromide” (Text word) OR “Anisodine” (Text word) AND [“Acute ischemic stroke” (Text Word) OR “ischemic stroke” (MESH) OR “brain ischemia” (MESH) OR “stroke” (MESH) OR “cerebral infarction” (MESH) OR “Cerebrovascular ischemia” (Text word) OR “Infarction, Anterior Cerebral Artery” (Text word) OR “Infarction, Middle Cerebral Artery” (Text word) OR “Infarction, Posterior Cerebral Artery” (Text word) OR “Apoplexy” (Text word)]. Potential studies in the reference lists of valid studies were also considered as information sources.

### 2.3 Inclusion and exclusion criteria

The inclusion criteria were as follows: 1) patients with AIS, regardless of age, gender, and disease stage; 2) parallel RCTs of Ani injection for AIS patients published in English or Chinese databases; 3) control group treated with regular therapies, while Ani injection was not applied in the control group; and 4) the trial groups were treated with Ani injection, used alone or in combination with the same regular therapies used in the control groups, regardless of the dose or duration of administration.

The exclusion criteria were as follows: 1) cerebral hemorrhage in patients with AIS; 2) reviews, letters, conference reports, cohort studies, case reports, cross-over studies, and animal studies; 3) duplicate studies or those with no comparison group; 4) literature without essential information or unable to obtain the related data; and 5) the trial group underwent acupuncture.

### 2.4 Outcome measures

In this systematic review and meta-analysis, the primary outcomes were as follows: National Institutes of Health Stroke Scale (NIHSS), modified Rankin Scale (mRS), and Barthel Index (BI). The secondary outcomes were computed tomography parameters (CTP), effective rate, and adverse events.

### 2.5 Study selection and data extraction

All electronic bibliographic databases mentioned above were scanned with a pre-designed search strategy. Duplicate articles were removed first. Next, two independent reviewers reviewed the titles and abstracts of the studies to select appropriate studies according to the eligibility criteria. The full texts of the selected studies were downloaded for further assessment. Three initial articles were used as a pilot to establish a standard extraction form, which contains the following domains: study information (title, first author, language, magazine, and year of publication), participant information (e.g., age, sex ratio, sample size, and disease course), intervention information (e.g., type, duration, frequency, and dose of treatment in the trial and control groups), and outcome indexes (primary outcomes and secondary outcomes). Reasons for the exclusion of ineligible studies were identified and recorded. Available data were extracted by two independent reviewers from the full texts. The two reviewers addressed disagreements through discussion or via consultation with a third reviewer.

### 2.6 Quality assessment

The Cochrane Collaboration Risk of Bias Tool in the Cochrane Handbook for Systematic Reviews of Interventions (Higgins et al., 2022) was used by two independent researchers to evaluate the methodological quality. According to the Cochrane Handbook, the risk of bias assessment was divided into seven domains: random sequence generation (selection bias), allocation concealment (selection bias), blinding of participants and personnel (performance bias), blinding of outcome assessment (detection bias), incomplete outcome data (attrition bias), selective reporting (reporting bias), and other bias. Each domain in the included RCTs was marked according to a low risk of bias, high risk of bias, or an unclear risk of bias. Disagreement between the two researchers was arbitrated by a third researcher.

### 2.7 Statistical analysis

The RevMan 5.3 software was used for meta-analysis. The relative risk (*RR*) was used as the effect index for the dichotomous variables, and the mean differences (*MD*) or standardized mean difference (*SMD*) were used as the effect index for continuous variables. The confidence interval (*CI*) of each effect index was set to 95%. The *I* ^*2*^ statistic was adopted to assess the heterogeneity. If *I* ^*2*^ > 50%, there was heterogeneity between the studies, and the random-effect model was selected; otherwise, the fixed-effect model was utilized. The heterogeneity was explained by sensitivity analysis or subgroup analysis. In addition, descriptive analysis was performed if the clinical data provided by the included studies were incomplete and could not be systematically evaluated. Potential publication bias was evaluated through a funnel plot.

## 3 Results

### 3.1 Study selection

**FIGURE 1:**
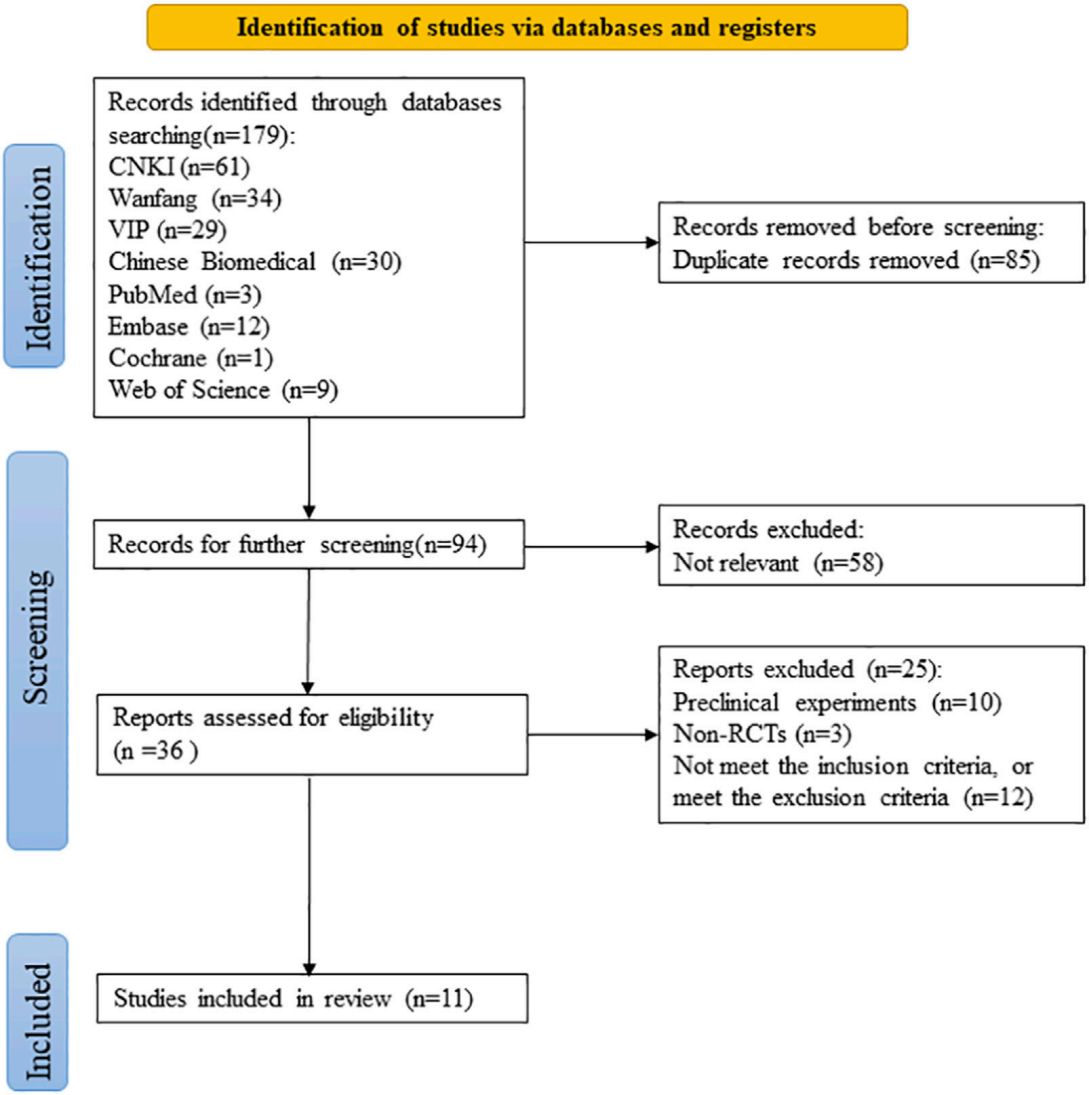
PRISMA flowchart for literature screening.

The PRISMA flowchart of the literature screening process is presented in Figure 1. Initially, according to the search strategy, a total of 179 studies were obtained through retrieval from multiple databases; after the deletion of 85 duplicate publications, the remaining 94 articles were screened. After examination of the titles and abstracts, 58 irrelevant studies were removed. The full texts of the 36 remaining articles were assessed for eligibility. Preclinical studies (*n* = 10), non-RCTs (*n* = 3), and studies that did not meet the inclusion or met the exclusion criteria (*n* = 12) were excluded. Finally, 11 studies were included in the meta-analysis (Zou et al., 2018; Wang et al., 2020; Dong et al., 2021; Li et al., 2021; Zhang et al., 2021; Jiang et al., 2022; Kang, 2022; Zhang, 2022; Zhang et al., 2022; Zhou, 2022; Yan et al., 2023).

### 3.2 Study characteristics

A total of 1,337 patients with AIS were included in the meta-analysis, including 668 patients in the trial group and 669 patients in the control group. All included studies were conducted in China. The sample size in each included study ranged from 21 to 193. The shortest treatment duration of an Ani injection was 7 days, and the longest was 30 days. The time period from AIS symptom onset to hospital admission was ≤72 h in all studies. Regarding the outcome measurements, the NIHSS was adopted in all studies (Zou et al., 2018; Wang et al., 2020; Dong et al., 2021; Li et al., 2021; Zhang et al., 2021; Jiang et al., 2022; Kang, 2022; Zhang, 2022; Zhang et al., 2022; Zhou, 2022; Yan et al., 2023), five studies used the mRS (Wang et al., 2020; Dong et al., 2021; Kang, 2022; Zhang, 2022; Zhang et al., 2022), four studies used the BI (Zou et al., 2018; Jiang et al., 2022; Zhang et al., 2022; Zhou, 2022), three studies reported the CTP (Zou et al., 2018; Zhang et al., 2022; Zhou, 2022), two studies mentioned the effective rate (Kang, 2022; Yan et al., 2023), and adverse events were described in seven studies (Zou et al., 2018; Dong et al., 2021; Li et al., 2021; Zhang et al., 2021; Jiang et al., 2022; Zhang, 2022; Zhang et al., 2022), while three studies reported the adverse rate (Dong et al., 2021; Li et al., 2021; Zhang et al., 2021). The characteristics of the included studies are presented in Table 1.

**TABLE 1:** Characteristics of included studies.

Study	Group	Number (M/F)	Age, years	Timeline of onset to initiation of therapy	Intervention and treatment duration	Outcomes
Zou et al. (2018)	Trial	43 (23/20)	65.32 ± 9.12	8.21 ± 1.32 (h)	CT + Ani intravenous, 2 mg daily for 14 days	①③④⑥
	Control	43 (21/22)	66.13 ± 10.31	7.96 ± 1.15 (h)	CT	
Wang et al. (2020)	Trial	35 (14/21)	68.2 ± 11.1	7–72 h	CT + Ani intravenous, 1 mg daily for 7 days	①②
	Control	35 (16/19)	72.7 ± 11.5		CT	
Zhang et al. (2021)	Trial	42 (23/19)	53.86 ± 3.19	NR	CT + Ani intravenous, 2 mg daily for 14 days	①⑥
	Control	42 (20/22)	52.71 ± 3.52		CT	
Li et al. (2021)	Trial	60 (38/22)	59.28 ± 11.82	10.82 ± 6.43 (h)	CT for 14 days + Ani intravenous, 2 mg daily for 14 days	①⑤⑥
	Control	60 (33/27)	60.04 ± 11.96	10.45 ± 6.29 (h)	CT for 14 days	
Dong et al. (2021)	Trial	100	63.22 ± 8.55	18.87 ± 18.95 (h)	CT + Ani intravenous, 2 mg daily for 14 days	①②⑥
	Control	101	64.25 ± 9.54	18.26 ± 17.63 (h)	CT	
Zhou (2022)	Trial	57 (30/27)	71.12 ± 2.74	≤24 h	CT for 90 days + Ani intravenous, 4 mg daily for 14 days	①③④
	Control	57 (31/26)	71.35 ± 2.65		CT for 90 days	
Zhang et al. (2022)	Trial	21	18–80	≤4.5 h	CT + Ani intravenous, 2 mg daily for 7∼14 days	①②③④⑥
	Control	21			CT	
Jiang et al. (2022)	Trial	193	63.43 ± 10.09	49.33 ± 27.42 (h)	CT + Ani intravenous, 1∼2 mg daily for 10∼14 days	①③⑥
	Control	193			CT	
Zhang et al. (2022)	Trial	16 (11/5)	50.14 ± 7.92	≤72 h	CT + Ani intravenous, 2 mg daily for 14 days	①②⑥
	Control	16 (10/6)	51.26 ± 9.35		CT	
Kang (2022)	Trial	71	NR	≤24 h	CT for 30 days + Ani intravenous, 2 mg daily for 30 days	①②⑤
	Control	71			CT for 30 days	
Yan et al. (2023)	Trial	30 (15/15)	62.8 ± 14.8	49.1 ± 27.8 (h)	CT + Ani intravenous, 2 mg daily for 14 days	①⑤
	Control	30 (17/13)	61.7 ± 15.6	48.3 ± 26.9 (h)	CT	

Note: M, male; F, female; NR, not reported; CT, conventional therapy; Ani, anisodine hydrobromide. Outcome indicators (① National Institutes of Health Stroke Scale; ② modified Rankin Scale; ③ Barthel Index; ④ CT, parameters; ⑤ clinical efficacy; ⑥ adverse events).

### 3.3 Risk of bias of the included studies

**FIGURE 2:**
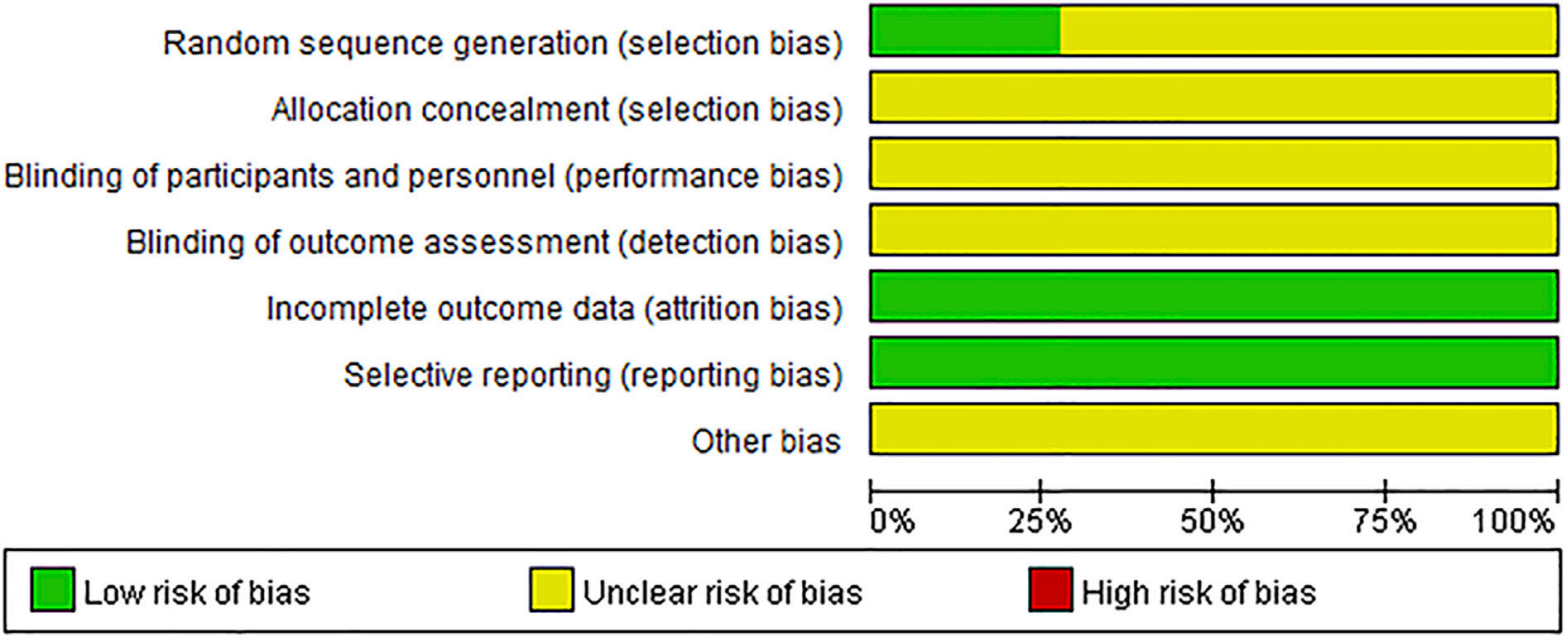
Risk of bias graph.

**FIGURE 3:**
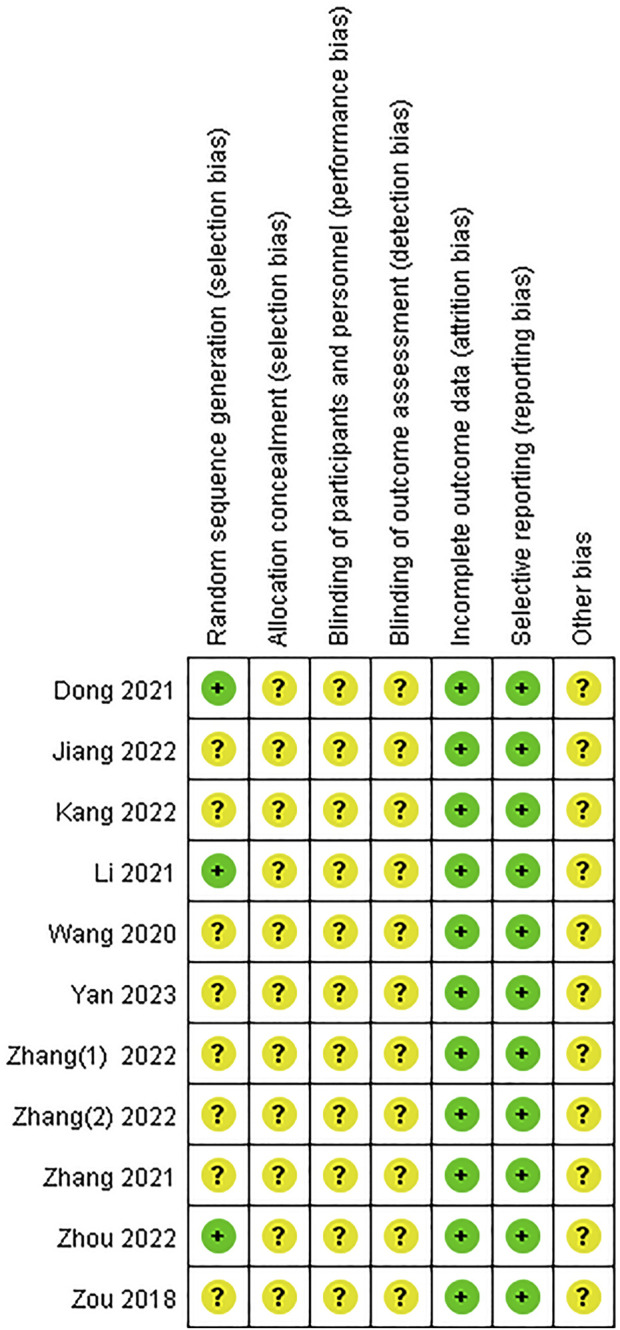
Risk of bias summary.

The assessment of bias risk of each eligible study was performed according to the Cochrane bias risk tool. Nine of the included studies mentioned grouping by a random method, three of which specified that a random sequence was generated through the random number table method (Dong et al., 2021; Li et al., 2021; Zhou, 2022). None of these studies referred to information on allocation concealment, blinding of participants and personnel, or blinding of the outcome assessment; therefore, all the studies were rated as having an unclear risk of bias in these three sections. All other bias evaluation risks were unclear. The results of the risk of bias assessment are presented in Figures 2, 3. See the supplementary document of Supplementary Material for rating bias (Supplementary Table S1).

### 3.4 Outcome measures

#### 3.4.1 National Institutes of Health Stroke Scale

**FIGURE 4:**
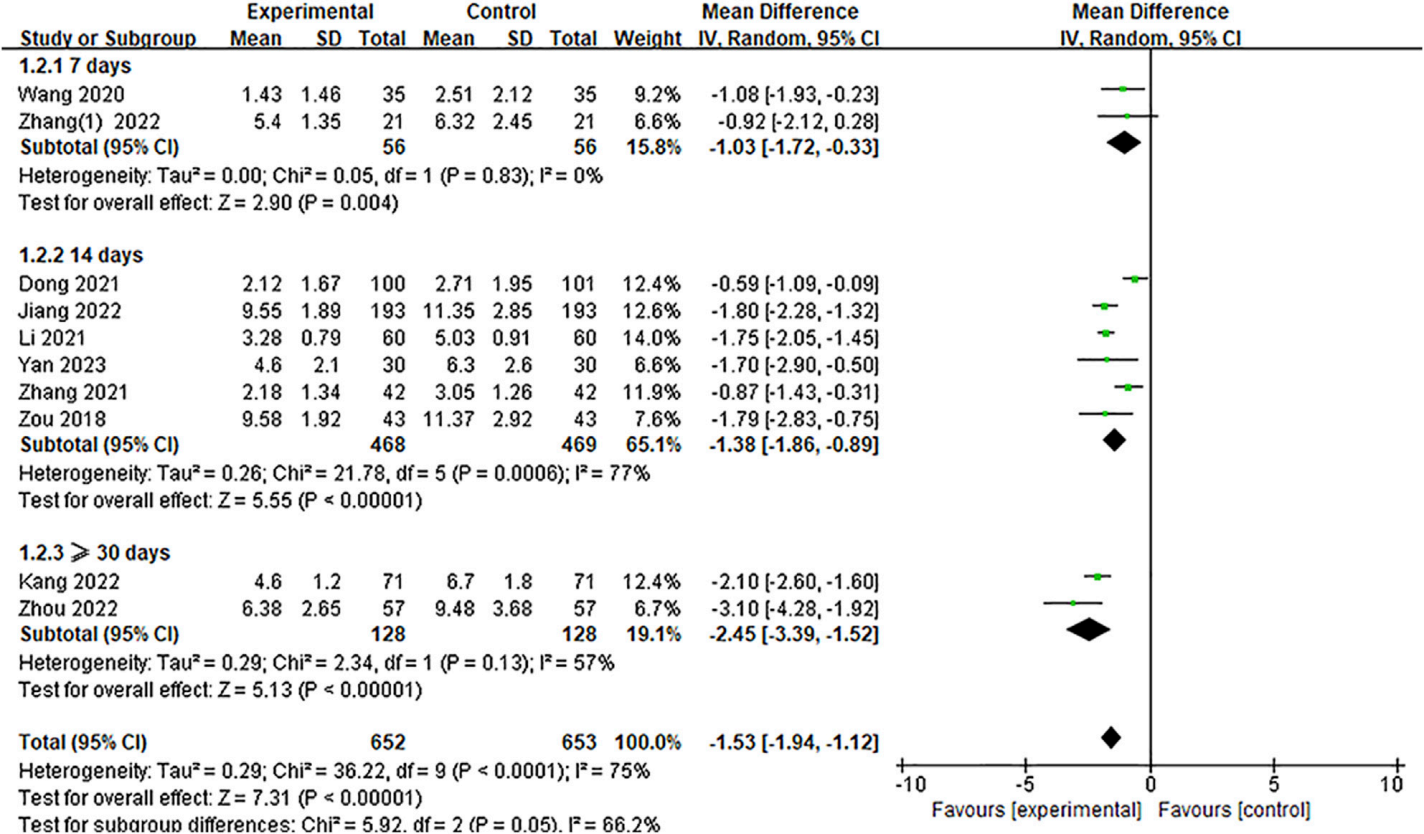
A meta-analysis of the NIHSS.

Eleven studies included the NIHSS score, of which one study (Zhang, 2022) did not report the post treatment NIHSS score; this data could not be extracted from the existing information. Thus, ten articles (Zou et al., 2018; Wang et al., 2020; Dong et al., 2021; Li et al., 2021; Zhang et al., 2021; Jiang et al., 2022; Kang, 2022; Zhang et al., 2022; Zhou, 2022; Yan et al., 2023) with 1,305 participants were included in the meta-analysis regarding the NIHSS score. The heterogeneity test results showed that *p* < 0.0001, *I* ^*2*^ = 75%, and there was significant heterogeneity among the studies. Therefore, a random-effect model was adopted. The pooled results of the post-treatment NIHSS score indicated that compared with the control group, Ani injection could significantly reduce the NIHSS score after treatment [MD = −1.53, 95%CI = (−1.94, −1.12), *p* < 0.00001], as shown in Figure 4.

In these studies, the NIHSS score was evaluated at different treatment time periods, which ranged from 7 days to 90 days of treatment. Therefore, we used treatment duration to conduct further NIHSS evaluations (7 days, 14 days, and ≥30 days) as the criteria for the subgroup analysis. The results of the subgroup analysis are shown in Figure 4. It can be clearly seen that at the different time periods of 7 days, 14 days, and ≥30 days for implementing the NIHSS assessment, the NIHSS score of the experimental group was significantly lower than that of the control group [MD = −1.03, 95%CI = (−1.72, −0.33), *p* = 0.004; MD = −1.38, 95%CI = (−1.86, −0.89), *p* < 0.00001; MD = −2.45, 95%CI = (−3.39, −1.52), *p* < 0.00001, respectively]. The result of the subgroup differences (*p* = 0.05, *I* ^*2*^ = 66.2%) indicated that this subgrouping factor might be a source of heterogeneity in the overall meta-analysis regarding the NIHSS score.

#### 3.4.2 Modified Rankin Scale

**FIGURE 5:**
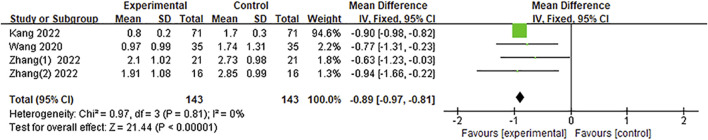
A meta-analysis of mRS.

Five studies reported the mRS score; however, the data could not be extracted from one study (Dong et al., 2021) due to the provided data being dichotomous. Thus, a total of four studies (Wang et al., 2020; Kang, 2022; Zhang, 2022; Zhang et al., 2022) were included. The results of the heterogeneity test demonstrated that *p* = 0.81 and *I* ^*2*^ = 0%; no significant heterogeneity was observed. Using the fixed-effect model, the results of the meta-analysis showed that the mRS score in the experimental group was significantly lower than that of the control group [MD = −0.89, 95%CI = (−0.97, −0.81), *p* < 0.00001], as shown in Figure 5.

#### 3.4.3 Barthel index

**FIGURE 6:**
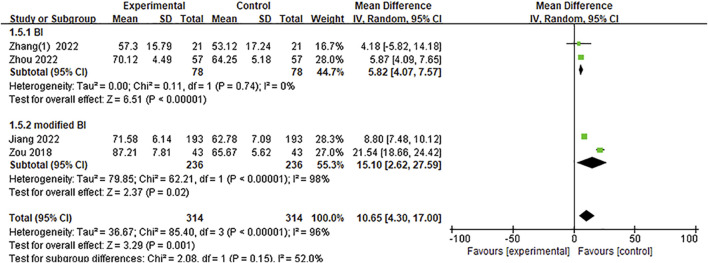
A meta-analysis of BI.

Four studies (Zou et al., 2018; Jiang et al., 2022; Zhang et al., 2022; Zhou, 2022) included the BI score. A meaningful increasing effect of Ani treatment was observed with the BI score level from the meta-analysis [MD = 10.65, 95%CI = (4.30, 17.00), *p* = 0.001]. Meanwhile, a significance between heterogeneity was observed (*p* < 0.00001, *I* ^*2*^ = 96%); thus, a random-effect model was adopted for the meta-analysis (Figure 6). The test for subgroup differences between trials that adopted the BI measurement [two RCTs (Zhang et al., 2022; Zhou, 2022)] [MD = 5.82, 95%CI = (4.07, 7.57), *p* < 0.00001] and modified BI measurement [two RCTs (Zou et al., 2018; Jiang et al., 2022)] [MD = 15.10, 95%CI = (2.62, 27.59), *p* = 0.02] was non-significant (*p* = 0.15, *I* ^*2*^ = 52%) (Figure 6).

#### 3.4.4 CT parameters

**FIGURE 7:**
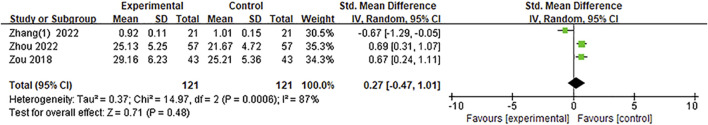
A meta-analysis of rCBF.

**FIGURE 8:**
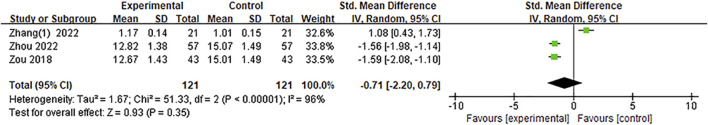
A meta-analysis of rMTT.

**FIGURE 9:**
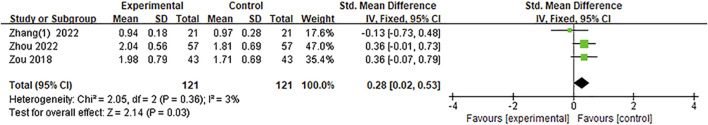
A meta-analysis of rCBV.

**FIGURE 10:**
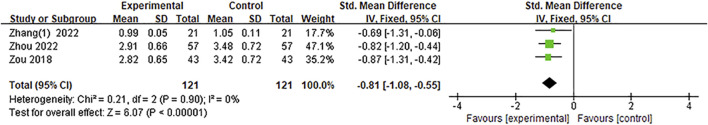
A meta-analysis of rTTP.

Three studies (Zou et al., 2018; Zhang et al., 2022; Zhou, 2022) reported the CTP, including relative cerebral blood flow (rCBF), relative cerebral blood volume (rCBV), relative time to peak (rTTP), and relative mean transit time (rMTT). The SMD was used as a summary statistic due to the consistency of the units in these studies being unclear. The pooled results indicated no statistically significant differences in the rCBF [SMD = 0.27, 95%CI = (−0.47, 1.01), *p* = 0.48] and rMTT [SMD = −0.71, 95%CI = (−2.20, 0.79), *p* = 0.35] between the Ani injection and the conventional therapy group and showed large heterogeneity (*p* = 0.0006, *I* ^*2*^ = 87%; *p* < 0.00001, *I* ^*2*^ = 96%, respectively) (Figures 7, 8). The effect of Ani injection on rCBF and rMTT, however, was not substantial. After sensitivity analysis by deleting one study (Zhang et al., 2022), the overall effect of Ani injection on rCBF and rMTT was significantly changed [SMD = 0.68, 95%CI = (0.40, 0.97), *p* < 0.00001; SMD = −1.57, 95%CI = (−1.89, −1.25), *p* < 0.00001, respectively]. Heterogeneity in both outcomes was also significantly reduced to 0%, which suggested that this study (Zhang et al., 2022) might be the source of the heterogeneity of the rCBF and rMTT data. Both the rCBV and rTTP levels were significantly changed by Ani injection therapy [SMD = 0.28, 95%CI = (0.02, 0.53), *p* = 0.03; SMD = −0.81, 95%CI = (−1.08, −0.55), *p* < 0.00001, respectively] without between-study heterogeneity (*p* = 0.36, *I* ^*2*^ = 3%; *p* = 0.90, *I* ^*2*^ = 0%, respectively) (Figures 9, 10).

#### 3.4.5 Clinical efficacy

**FIGURE 11:**
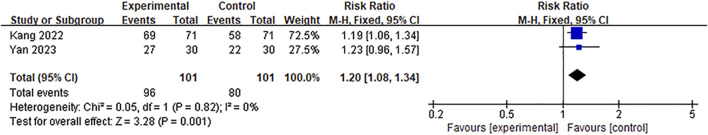
A meta-analysis of clinical efficacy.

Two studies (Kang, 2022; Yan et al., 2023) reported the clinical effective rate, which was evaluated according to the NIHSS score for stroke patients. The heterogeneity test results showed that there was no significant heterogeneity among these studies (*p* = 0.82, *I* ^*2*^ = 0%); therefore, the fixed-effect model was adopted. The pooled results showed that the effective rate of the Ani injection-treated group was significantly better than that of the conventional therapy group [RR = 1.2, 95%CI = (1.08, 1.34), *p* = 0.001] (Figure 11).

#### 3.4.6 Adverse events

A total of seven articles (Zou et al., 2018; Dong et al., 2021; Li et al., 2021; Zhang et al., 2021; Jiang et al., 2022; Zhang, 2022; Zhang et al., 2022) recorded adverse reactions, of which two (Zhang, 2022; Zhang et al., 2022) reported that no adverse events occurred during treatment and two (Zou et al., 2018; Jiang et al., 2022) reported mild side effects, including dry mouth and facial flushing; these symptoms had completely disappeared after slowing down the drip rate.

**FIGURE 12:**
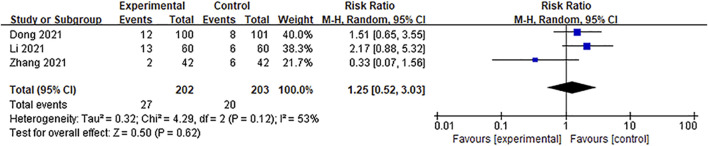
A meta-analysis of the rate of adverse events.

The other three articles (Dong et al., 2021; Li et al., 2021; Zhang et al., 2021) reported the incidence of adverse reactions. Using the random-effect model (*p* = 0.12, *I* ^*2*^ = 53%) for the meta-analysis, the pooled results showed that there were no significant differences between the Ani injection supplemental group and the control group [RR = 1.25, 95%CI = (0.52, 3.03), *p* = 0.62] (Figure 12). Dong et al. (2021) observed 12 cases of dry mouth and facial flushing in the experimental group and 8 cases of nausea and vomiting in the control group. Zhang et al. (2021) reported 6 cases of nausea, vomiting, dry mouth, and facial flushing in the control group and 2 cases of nausea and vomiting in the experimental group. Li et al. (2021) found 2 cases of elevated alanine aminotransferase (ALT), 2 cases of dizziness, 3 cases of weakness, and 6 cases of gastrointestinal reactions in the experimental group and 1 case of elevated ALT, 1 case of weakness, and 4 cases of gastrointestinal reactions in the control group. Table 2 presents the adverse reactions of the involved studies.

**TABLE 2:** Summary of adverse events of the involved studies.

Study	Group	Number	Number of adverse reactions	Adverse reactions
Zou et al. (2018)	Trial	43	NR	Mild flushed face, dry mouth, dizziness
	Control	43	NR	NR
Zhang et al. (2021)	Trial	42	2	Nausea, vomiting
	Control	42	6	Flushed face, dry mouth, nausea, vomiting
Li et al. (2021)	Trial	60	13	Elevated alanine aminotransferase, dizziness, fatigue, gastrointestinal reactions
	Control	60	6	Elevated alanine aminotransferase, fatigue, gastrointestinal reactions
Dong et al. (2021)	Trial	100	12	Flushed face, dry mouth
	Control	101	8	Nausea, vomiting
Zhang (2022)	Trial	21	NR	No adverse reactions occurred
	Control	21	NR	No adverse reactions occurred
Jiang et al. (2022)	Trial	193	NR	Mild flushed face, dry mouth, bitter mouth, loss of appetite
	Control	193	NR	Mild flushed face, dry mouth, bitter mouth, loss of appetite
Zhang et al. (2022)	Trial	16	NR	No adverse reactions occurred
	Control	16	NR	No adverse reactions occurred

Note: NR, not reported.

### 3.5 Publication bias

**FIGURE 13:**
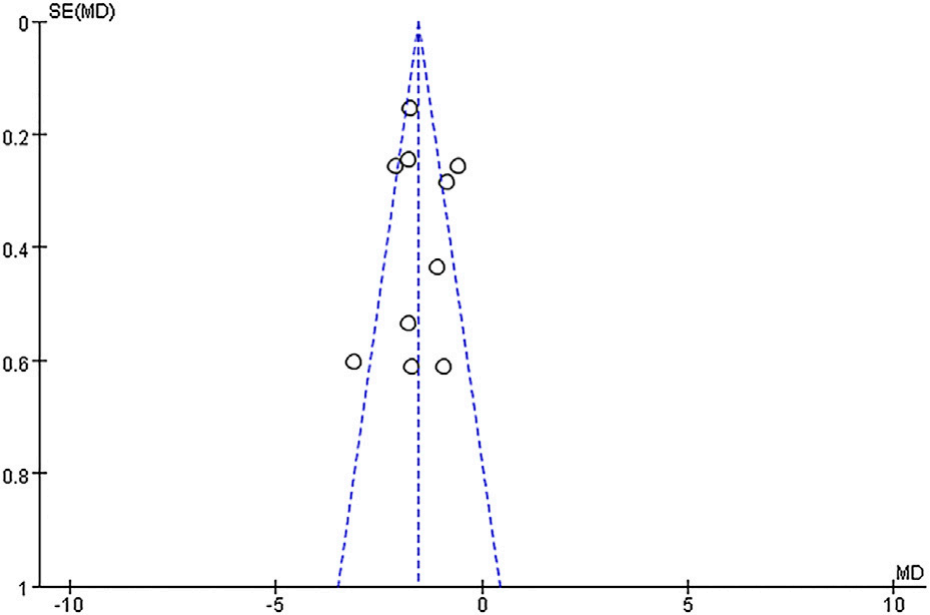
Funnel plot for the publication bias of the NIHSS.

A funnel plot was conducted to assess the publication bias of 10 trials or more. Thus, the 10 included studies that included available NIHSS score data were used for publication bias assessment, as shown in Figure 13. The shape of the funnel plot of the NIHSS showed the moderate symmetry between the included studies, which indicated that the potential of publication bias was low.

## 4 Discussion

### 4.1 Summary of findings

A total of 11 RCTs were included in this meta-analysis. Combined with conventional therapy, Ani injection was used to treat 1,337 patients with AIS. The NIHSS, mRS, BI, CTP, effective rate, and adverse events were evaluated in the analysis. According to the findings, the NIHSS score of Ani injection therapy was much lower than that of conventional therapy alone. Other primary indicators showed that Ani injection significantly reduced the mRS score and increased the BI score. The secondary outcome indicators revealed that treatment with Ani injection increased the rCBV, reduced the rTTP, and improved the clinical efficacy, with significant differences observed. The pooled analysis of the included studies failed to identify a significant change in the rCBF, rMTT, and rate of adverse reactions.

Subgroup analyses indicated that at the different time periods of 7 days, 14 days, and ≥30 days for implementing the NIHSS assessment, the NIHSS score of the Ani treatment group was considerably decreased. Furthermore, the subgrouping factor might be a source of significant heterogeneity for the NIHSS. Subgroup analyses on the BI score based on the BI assessment method (original BI and modified BI) suggest that regardless of the BI assessment method used, Ani treatment exhibits an advantage in significantly increasing the BI score. However, the subgroup analysis of BI did not identify the source of heterogeneity; significant heterogeneity may be associated with factors such as small sample sizes and few included studies.

A sensitivity analysis of rCBF and rMTT suggested that the pooled results are not robust and that the study of Zhang et al. (2022) might be the source of rCBF and rMTT heterogeneity.

In the risk of bias section, the quality assessment of the current included studies showed that allocation concealment, blinding of participants and employees, and blinding of the outcome assessment, as well as other forms of bias, were not disclosed in any of the included studies, which suggested that the certainty of evidence in the included RCTs was not high. Consequently, the results of the meta-analysis may be influenced, and our findings based on the current evidence should be considered carefully in the clinic. More precise RCTs are needed to further validate the curative effect of Ani injection in patients with AIS.

### 4.2 Interpretation

Traditional Chinese herbal medicines have a long history of clinical application in treating various vascular diseases, with distinctive theories and rich practices (Hung et al., 2015; Hao et al., 2017). Products from traditional Chinese medicinal herbs have been widely described in various ancient medicine systems for treating ischemic stroke, myocardial infarction, and so on (Hung et al., 2015). Anisodine is one of the most important ingredients of the tropane-type alkaloids extracted from the traditional folk medicinal herb *A. tanguticus*, with significant biological activities for promoting blood circulation and removing blood stasis (Meng et al., 2023). Pharmaceutical products containing anisodine are frequently used in the clinic for the treatment of vascular diseases, including ischemic stroke (Zou et al., 2018), retinal artery occlusion (Wu et al., 2016), ischemic optic neuropathy (Zhang et al., 2019), and cerebral small vessel disease (Gui et al., 2019). Ani injection has been developed for improved chemical stability and is a promising treatment for AIS.

Poor perfusion of brain tissue caused by the abrupt interruption or reduction of cerebral blood flow is the etiology of AIS, which can then induce ischemic hypoxic necrosis, clinically manifesting as different degrees of neurological impairment (Brott and Bogousslavsky, 2000). The molecular mechanism of AIS can be summarized as a complex series of ischemic cascades, characterized by cellular bioenergetic failure, excitotoxicity, excessive intraneuronal accumulation of Na^+^, Cl^−^, and Ca^2+^, oxidative damage, inflammatory reaction, mitochondrial injury, and, finally, cell death (Rosenblum, 1997; Brouns and De Deyn, 2009). Guidelines for the management of AIS have been reported by various countries (Swain et al., 2008; Di et al., 2019; Powers et al., 2019); the fundamental goals of the intervention have been focused on restoring or increasing the blood supply to the brain and blockading or slowing of the cerebral ischemic cascade (Olsen et al., 1983; Escuret, 1995; Brott and Bogousslavsky, 2000). The conventional therapy adopted in the 11 included studies varied across different care settings, including general management (such as respiratory and oxygen intake, cardiac monitoring and cardiac disease management, temperature control, blood pressure control, blood sugar control, and nutritional support, etc.) and specific treatment (thrombolysis, antiplatelet drugs, anticoagulants, statins, defibrase, and diuretics, etc.). Nevertheless, the narrow treatment window and hemorrhagic complications have limited the utilization and therapeutic effect of conventional therapy.

Recent studies have revealed that in addition to the recanalization of the large cerebral vessels, the restoration of normal vasomotor function around the ischemic area, the improvement in micro-perfusion, and neuronal cell protection are crucial for the treatment of cerebral infarction and are closely related to the prognosis of AIS (Tuttolomondo et al., 2009; Shuaib et al., 2011; Bang et al., 2015). As a central muscarinic cholinergic antagonist, Ani can effectively relieve vasospasm, open closed arterioles and the anterior capillary sphincter, and restore the perfusion of brain tissue (Wang et al., 2018). Research has demonstrated that following Ani administration, the microvascular autonomic motion reappears in the small intestinal wall micro-artery ischemia model, with a significant increase in microvascular amplitude, blood velocity, and flow (Zhang et al., 2019). Through the establishment of a hypoxia/reoxygenation (H/R)-induced brain microvascular endothelial cell injury model, Ani injection has been shown to suppress H/R-induced hypoxia-inducible transcription factor 1(HIF-α) over-expression, nitric oxide (NO), and reactive oxygen species (ROS) production, and all these effects were dependent on M4-AchR (Zeng et al., 2021). Additionally, Ani has multiple non-cholinergic effects, including cell protective effects, autophagy (Chen et al., 2017b), attenuating neuronal cell death and apoptosis (Chen et al., 2017d), alleviating oxidative stress damage and decreasing Ca^2+^ accumulation (Chen et al., 2017; Wang et al., 2017; Chen et al., 2017e), and inhibiting membrane lipid peroxidation (Zhao and Chen, 2010), thereby alleviating cell damage caused by ischemia and hypoxia. Further studies have found that Ani can decrease the Longa rodent stroke scores and cerebral infarction area in middle cerebral artery occlusion (MCAO) rats (Chen et al., 2017c). Moreover, the underlying mechanism of the effect of Ani on AIS can also be attributed to the ability to improve hemorheology and resist platelet aggregation so as to improve cerebral microcirculation disorders (Xu et al., 2020).

### 4.3 Strengths and limitations

In short, based on current evidence, Ani injection therapy was found to be effective and safe in patients with AIS. The positive effect of Ani injection may be attributed to the ability of Ani to penetrate the blood–brain barrier and act as a non-specific muscarinic cholinergic receptor antagonist, competing with acetylcholine in the central nervous system, resulting in increased cerebral blood supply and neuroprotection effects (Liu et al., 2020; Jiang et al., 2022). The results of this meta-analysis demonstrated that the efficacy of Ani injection in the treatment of AIS was superior to that of conventional therapy; however, several limitations still exist. First, the sample size of the included RCTs was small, so the subgroup analysis and publication bias assessment could not be conducted for all indicators, which may have affected the accuracy and reliability of the results. Second, all trials lacked a precise description of the allocation concealment and blinding methods (for participants, personnel, and outcome assessments); the description of random sequence generation was also missed in some studies. As a consequence, the general methodological quality of the studies was not satisfactory. Third, the longest intervention duration in all the articles was 90 days, and there was a lack of long-term follow-up visits for more than 90 days after treatment, which was insufficient to assess the long-term impact of Ani therapy on the health of patients. Additionally, all the included RCTs were conducted in China; therefore, it is necessary to utilize multi-regional clinical trials for Ani treatment evaluation in different regions of the world in the future to provide strong evidence for the efficacy and safety of Ani treatment in patients with AIS.

## 5 Conclusion

Taken together, the meta-analysis results from the included RCTs revealed that Ani injection is effective and safe in the treatment of patients with AIS, with positive impacts on the NIHSS, mRS, BI, rCBV, rTTP, and clinical efficacy. However, due to limitations in the number and quality of included studies, more multi-center, large-sample, high-quality RCTs are needed for further verification of the efficacy and safety of Ani injection in treating AIS.

## Data Availability

The original contributions presented in the study are included in the article/Supplementary Material, further inquiries can be directed to the corresponding authors.
