# Supplementary material for: Efficacy and safety of anisodine hydrobromide injection for acute ischemic stroke: a systematic review and meta-analysis
Source: Front Pharmacol. 2023 Nov 15;14:1290755. doi: 10.3389/fphar.2023.1290755 (PMC10684921; doi:10.3389/fphar.2023.1290755)
Supplement: Supplementary file 1 [file Table1.DOCX]

Supplementary Table S1. Supporting information for rating bias

| **Included studies** | **Random sequence generation** | **Allocation concealment** | **Blinding of participants and personnel** | **Blinding of outcome assessment** | **Incomplete outcome data** | **Selective reporting** | **Other bias** |
| --- | --- | --- | --- | --- | --- | --- | --- |
| Dong 2021 | Random number table method was used for generating random sequence; thus, it is low risk of bias. | The related information in the text was not mentioned; thus, it is unclear of bias. | The related information in the text was not mentioned; thus, it is unclear of bias. | The related information in the text was not mentioned; thus, it is unclear of bias. | There is no missing data in the text; thus, it is low risk of bias. | All the pre-specified outcome indicators in the research methods have been reported; thus, it is low risk of bias. | The related information in the text was not mentioned; thus, it is unclear of bias. |
| Jiang 2022 | The article only reported compliance with the principle of random allocation, but did not specify a specific method; thus, it is unclear of bias. | The related information in the text was not mentioned; thus, it is unclear of bias. | The related information in the text was not mentioned; thus, it is unclear of bias. | The related information in the text was not mentioned; thus, it is unclear of bias. | There is no missing data in the text; thus, it is low risk of bias. | All the pre-specified outcome indicators in the research methods have been reported; thus, it is low risk of bias. | The related information in the text was not mentioned; thus, it is unclear of bias. |
| Kang 2022 | The article only reported compliance with the principle of random allocation, but did not specify a specific method; thus, it is unclear of bias. | The related information in the text was not mentioned; thus, it is unclear of bias. | The related information in the text was not mentioned; thus, it is unclear of bias. | The related information in the text was not mentioned; thus, it is unclear of bias. | There is no missing data in the text; thus, it is low risk of bias. | All the pre-specified outcome indicators in the research methods have been reported; thus, it is low risk of bias. | The related information in the text was not mentioned; thus, it is unclear of bias. |
| Li 2021 | Random number table method was used for generating random sequence; thus, it is low risk of bias. | The related information in the text was not mentioned; thus, it is unclear of bias. | The related information in the text was not mentioned; thus, it is unclear of bias. | The related information in the text was not mentioned; thus, it is unclear of bias. | There is no missing data in the text; thus, it is low risk of bias. | All the pre-specified outcome indicators in the research methods have been reported; thus, it is low risk of bias. | The related information in the text was not mentioned; thus, it is unclear of bias. |
| Wang 2020 | The article only reported compliance with the principle of random allocation, but did not specify a specific method; thus, it is unclear of bias. | The related information in the text was not mentioned; thus, it is unclear of bias. | The related information in the text was not mentioned; thus, it is unclear of bias. | The related information in the text was not mentioned; thus, it is unclear of bias. | There is no missing data in the text; thus, it is low risk of bias. | All the pre-specified outcome indicators in the research methods have been reported; thus, it is low risk of bias. | The related information in the text was not mentioned; thus, it is unclear of bias. |
| Yan 2023 | The article only reported compliance with the principle of random allocation, but did not specify a specific method; thus, it is unclear of bias. | The related information in the text was not mentioned; thus, it is unclear of bias. | The related information in the text was not mentioned; thus, it is unclear of bias. | The related information in the text was not mentioned; thus, it is unclear of bias. | There is no missing data in the text; thus, it is low risk of bias. | All the pre-specified outcome indicators in the research methods have been reported; thus, it is low risk of bias. | The related information in the text was not mentioned; thus, it is unclear of bias. |
| Zhang (1) 2022 | The article only reported compliance with the principle of random allocation, but did not specify a specific method; thus, it is unclear of bias. | The related information in the text was not mentioned; thus, it is unclear of bias. | The related information in the text was not mentioned; thus, it is unclear of bias. | The related information in the text was not mentioned; thus, it is unclear of bias. | There is no missing data in the text; thus, it is low risk of bias. | All the pre-specified outcome indicators in the research methods have been reported; thus, it is low risk of bias. | The related information in the text was not mentioned; thus, it is unclear of bias. |
| Zhang (2) 2022 | The article only reported compliance with the principle of random allocation, but did not specify a specific method; thus, it is unclear of bias. | The related information in the text was not mentioned; thus, it is unclear of bias. | The related information in the text was not mentioned; thus, it is unclear of bias. | The related information in the text was not mentioned; thus, it is unclear of bias. | There is no missing data in the text; thus, it is low risk of bias. | All the pre-specified outcome indicators in the research methods have been reported; thus, it is low risk of bias. | The related information in the text was not mentioned; thus, it is unclear of bias. |
| Zhang 2021 | The article only reported compliance with the principle of random allocation, but did not specify a specific method; thus, it is unclear of bias. | The related information in the text was not mentioned; thus, it is unclear of bias. | The related information in the text was not mentioned; thus, it is unclear of bias. | The related information in the text was not mentioned; thus, it is unclear of bias. | There is no missing data in the text; thus, it is low risk of bias. | All the pre-specified outcome indicators in the research methods have been reported; thus, it is low risk of bias. | The related information in the text was not mentioned; thus, it is unclear of bias. |
| Zhou 2022 | Random number table method was used for generating random sequence; thus, it is low risk of bias. | The related information in the text was not mentioned; thus, it is unclear of bias. | The related information in the text was not mentioned; thus, it is unclear of bias. | The related information in the text was not mentioned; thus, it is unclear of bias. | There is no missing data in the text; thus, it is low risk of bias. | All the pre-specified outcome indicators in the research methods have been reported; thus, it is low risk of bias. | The related information in the text was not mentioned; thus, it is unclear of bias. |
| Zou 2018 | The article only reported compliance with the principle of random allocation, but did not specify a specific method; thus, it is unclear of bias. | The related information in the text was not mentioned; thus, it is unclear of bias. | The related information in the text was not mentioned; thus, it is unclear of bias. | The related information in the text was not mentioned; thus, it is unclear of bias. | There is no missing data in the text; thus, it is low risk of bias. | All the pre-specified outcome indicators in the research methods have been reported; thus, it is low risk of bias. | The related information in the text was not mentioned; thus, it is unclear of bias. |
